# Severe hyperemesis gravidarum affects offspring metabolism in childhood

**DOI:** 10.1186/1687-9856-2013-S1-O51

**Published:** 2013-10-03

**Authors:** Ahila Ayyavoo, Paul L Hofman, José GB Derraik, Sarah Mathai, Peter Stone, Frank Bloomfield, Wayne S Cutfield

**Affiliations:** 1 Liggins Institute, University of Auckland, Auckland, New Zealand; 2 National Research Centre for Growth and Development, University of Auckland, Auckland, New Zealand; 3 Department of Obstetrics and Gynaecology, Faculty of Medical and Health Sciences, University of Auckland, Auckland, New Zealand

## 

Hyperemesis gravidarum leads to alterations in maternal (and possibly fetal) nutrition throughout pregnancy, but there are no data on the long-term metabolic health outcomes in the offspring. We hypothesized that hyperemesis gravidarum could lead to fetal nutritional compromise or physiological stress, which may programme later metabolism and body composition.

Two groups of healthy pre-pubertal children born at term, aged 4–11 years were studied: offspring of mothers who suffered hyperemesis gravidarum (HG group; n=36) and controls (n=54). Recruited HG children were born to mothers admitted to hospital with metabolic disturbance during pregnancy. Following an overnight fast, a frequently sampled intravenous glucose tolerance test modified by insulin was performed, and insulin sensitivity was measured using Bergman’s minimal model. Other assessments included fasting lipid and hormonal profiles, as well as body composition using whole-body dual-energy x-ray absorptiometry. Data were analysed separately using linear mixed models, controlling for appropriate confounders. Data are expressed as mean±SEM.

Children born to mothers with severe HG had reduced S_I_ (10.4±0.6 vs 13.4±0.9 x10^-4^min^−1^·(mU/L); p=0.016), increased fasting insulin (6.5±0.6 vs 4.9±0.3 mIU/L; p=0.023), reduced IGFBP1 (13.0±1.3 vs 18.0±1.6 ng/ml; p=0.029) and IGFBP3 (3017±106 vs 3497±102 ng/ml; p=0.008) in comparison to controls. Baseline cortisol was higher in HG children (251±13 vs 218±11 nmol/l; p=0.007). DEXA-derived body composition was similar in HG and control groups.

Children born to mothers who experienced severe hyperemesis gravidarum were less insulin sensitive and had elevated baseline cortisol compared to controls. We postulate that severe hyperemesis gravidarum reduces insulin sensitivity in the offspring due to fetal programming of the fetal hypothalamic-pituitary-adrenal (HPA) axis. Long-term follow up of these offspring is essential to determine later risk of metabolic disease.

